# Preclinical models in head and neck squamous cell carcinoma

**DOI:** 10.1038/s41416-023-02186-1

**Published:** 2023-02-10

**Authors:** Patricia Chaves, María Garrido, Javier Oliver, Elisabeth Pérez-Ruiz, Isabel Barragan, Antonio Rueda-Domínguez

**Affiliations:** 1 Medical Oncology Service (Group of Translational Research in Cancer Immunotherapy), Regional and Virgen de la Victoria University Hospitals, Instituto de Investigación Biomédica de Málaga y Plataforma en Nanomedicina–IBIMA Plataforma Bionand, C/Marqués de Beccaría n°3, 29010 Málaga, Spain; 2 Group of Pharmacoepigenetics, Department of Physiology and Pharmacology, Karolinska Institutet, Stockholm, Sweden

**Keywords:** Oral cancer, Oral cancer

## Abstract

Head and neck cancer is the sixth most frequent cancer type. Drug resistance and toxicity are common challenges of the existing therapies, making the development of reliable preclinical models essential for the study of the involved molecular mechanisms as well as for eventual intervention approaches that improve the clinical outcome. Preclinical models of head and neck squamous cell carcinoma have been traditionally based on cell lines and murine models. In this review, we will go over the most frequently used preclinical models, from immortalised-cell and primary tumour cultures in monolayer or 3D, to the currently available animal models. We will scrutinise their efficiency in mimicking the molecular and cellular complexity of head and neck squamous cell carcinoma. Finally, the challenges and the opportunities of other envisaged putative approaches, as well as the potential of the preclinical models to further develop personalised therapies will be discussed.

## Introduction

Head and neck cancer (HNC) refers to a wide range of heterogeneous disorders that start in the head and neck area. It is the sixth most common malignancy worldwide and 90% of the cases are squamous (head and neck squamous cell carcinoma, HNSCC) [1, 2]. HNSCC occurs with the highest prevalence in elderly men exposed to smoking, alcohol abuse, Epstein-Barr (EB) virus infection (especially in nasopharyngeal carcinoma), and human papillomavirus (HPV) infection (especially in oropharyngeal cancer). More recently, the rising frequency of HPV-associated oropharyngeal cancer has considerably changed the epidemiology of HNSCC [3]. Surgical resection and other therapies including radiation, chemotherapy and immunotherapy have improved the life quality of the patients [4], but local bone invasion, distant metastasis and drug resistance are usual complications of this aggressive cancer, leading to low survival rates [1].

Unfortunately, it should be added that, compared to other malignancies, HNSCC has experienced little therapeutic development and very few drugs have been approved in the last decades. The use of biomarkers for the improvement of tumour staging, prognosis, and customised treatment has been hardly incorporated to routinely clinical use, mainly due to the high heterogeneity of HNSCC [5]. Thus, tailoring the treatment towards improving the clinical benefit, requires a better understanding of the mechanisms involved in HNSCC development and response to treatment through the study of models that closely resemble the in vivo process of the disease. In this review, we provide a comprehensive description of the available preclinical models, with focus on those most extensively used.

**Fig. 1:**
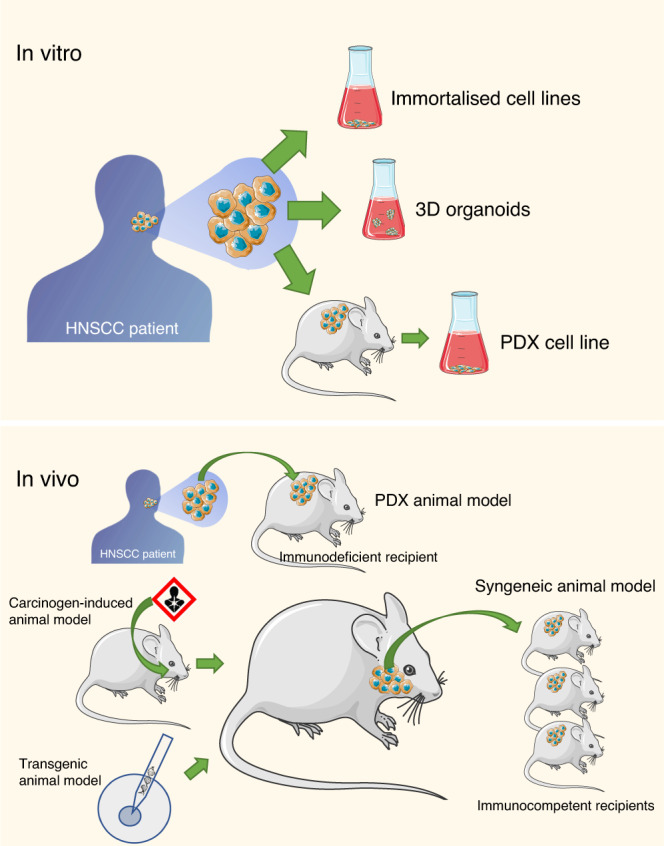
Main experimental approaches for generating preclinical models in HNSCC research. Above, in vitro preclinical models include immortalised cell lines, 3D organoids and PDX cell lines. Below, in vivo murine preclinical models include PDX, syngeneic, carcinogen induced and transgenic animal models.

Reliable preclinical models are a requirement for a priori testing the efficacy of novel therapeutic strategies including the evaluation of tailored therapies. They are also useful for generating diagnostic and monitoring biomarkers. Indeed, different model-building approaches result appropriate for specific purposes (Fig. 1). For example, HNSCC cell lines have been considered the most affordable method to understand certain molecular mechanisms involved in drug efficacy, whereas three-dimensional (3D) cultures that more clearly depict tumour tissue architecture and cellular environment and can be more informative for biomarker generation, are under promising development. Preclinical animal models, from xenograft implants to genetically modified mice, have been traditionally used to reproduce tumour initiation and progression and to test the efficacy of drugs. Here, we will analyse the advantages and disadvantages of using each of these preclinical models (Table 1), describe the methods that are most frequently used, and elaborate on the future perspectives in the field.

**Table 1:** Advantages and disadvantages of preclinical models in HNSCC.

Preclinical model		Advantages	Disadvantages	Reference
In vitro	General	Cell diversity is kept	No tumour microenvironment	[7, 8]
		Useful for mechanistic studies	Genomic instability	
		Inexpensive and fast	Poor predictor for clinical benefits	
	Immortalised-cell lines	Inexpensive	Genomic instability	[7–13]
		Easy maintenance	Clonal selection	
		Allow genetic modifications	Lack of reproducibility	
	PDXs	Keep genetic heterogeneity	More expensive and longer to develop than immortalised lines	[14–16]
		Allow genetic modifications	Mouse microenvironment	
	3D Spheroids and Organoids	Reproduce tumour structure	Still under development	[17, 18]
		Keeps genetic heterogeneity	Lack of ECM effect	
In vivo	General	Tumour microenvironment	Long-term experiments	[8, 28]
		Resemblance with human disease	No appropriate model for all purposes	
	PDX	Can lead to invasion and metastasis development	No immune-tumour interaction.	[46, 47]
		Maintains tumour histological and genetic features	Time-consuming and expensive	
	Carcinogen-induced	Resembles tumour initiation	Long term for lesion development	[69–71]
		Keeps genetic heterogeneity	Only for accessible body regions	
		Immune system is kept intact	Toxicity for operators	
	Transgenic	Control over gene expression	Time-consuming and expensive	[73, 74]
		Tumour development mechanism similar to human	Gene expression is altered	
		Allow study from initiation until progression	Unpredictability over tumour formation	

## HNSCC in vitro models

### History and origin of HNSCC cell lines

Cancer research has traditionally relied on in vitro preclinical models to study the effect of therapies and to facilitate diagnosis, being used as high-throughput screening platforms. Even today, when the high relevance of the cellular and molecular nature of the tumour microenvironment has been demonstrated, in vitro models are widely used for drug discovery, monitoring of disease progression and the design of tailored therapies. After numerous efforts, 2D models have evolved to more physiologically relevant 3D cell cultures and are still essential in cancer research, however, they still require further advances to help improve the rise of precision medicine [6].

### HNSCC cell lines

#### Immortalised-cell lines

Immortalised-cell lines have long been employed as in vitro models for cancer biology and pharmacogenomics research. Immortality is achieved by blocking the cell cycle checkpoint pathways using different protocols such as ectopic expression of telomerase, telomerase reverse transcriptase (TERT) or p53 and pRb-mutated genes [7, 8].

Among all HNSCC cell lines, 60% correspond to the oral cavity, as the primary treatment for these pathologies is surgery. Cell lines immortalised from pharyngeal tumours constitute 12% of the total, while 18% are derived from the larynx and just 3% from the nasal septum [9]. It is remarkable the fact that only six HPV + cell lines are currently available, most likely due to the very low success in cell line establishment. Although the cause of this phenomenon is yet unclear, the current situation raises the question of whether the HPV + cell lines that are usable are sufficient to investigate clinically relevant mechanisms or treatment responses. In addition, the fact that oropharyngeal tumours, the most frequent malignancies induced by HPV, are very rarely subject to primary surgery, also reduces the potential usability of this method [7].

HNSCC cell lines have been characterised at different levels in several studies. Barrentina et al. founded The Cancer Cell Line Encyclopedia (CCLE), a comprehensive database of human cancer diversity which also includes the molecular characterisation of 947 human cancer cell lines from 36 different tissue types, including 32 HNSCC cell lines. Garnett et al. systematically profiled the genome and pharmacologic response of more than 300 cell lines from which 21 were derived from HNSCC tumours, to identify drug sensitivity markers [10]. Also, Lepikhova et al. genetically profiled 45 human HPV-negative-HNSCC cell lines under the effect of 220 anticancer drugs that had been established at the Department of Otorhinolaryngology-Head and Neck Surgery, in Turku University Hospital [11], These and other studies show that *TP53, CDKN2A, CDKN2a(p14), SMAD4*, *PIK3CA, mTOR, NOTCH* or *EGFR* are the most common mutated genes in HNSCC cell lines [9–12].

Other providers of HNSCC cell lines are The National Cancer Institute (NCI) and the Hamon Cancer Center (HCC), which have a large collection of HNSCC cell lines accessible through the American Type Culture Collection (Manassas, VA, USA) [12]. Finally, the University of Michigan has a repository of squamous cell carcinoma cell lines with more than 120 HNSCC cell lines, including some HPV-positive ones [13]. Although the technical sophistication of the permanent culture of HNSCC cell lines has been substantially increased, immortalised-cell lines still have important limitations that compromise their use as models as well as their experimental reproducibility: (i) they do not properly reflect the histological nature of the tumour, (ii) they undergo clonal selection induced by selective survival pressures intrinsic to the culture conditions, and (iii) their genetic and epigenetic differences from the original tumours reduce their biological fidelity [6, 13].

#### PDXs cell lines

PDX-derived cell lines have been of widespread use in research only recently. As we will explain later, PDXs constitute an effective preclinical approach in clinical translational research because, unlike cell lines that grow in vitro under non-physiological conditions, they get established in vivo in a 3D microenvironment that is fully provided of nutrients, oxygen and communication with non-tumour cells such as host stromal and immune cells, so they closely resemble the heterogeneity and genetic characteristics of the human tumour. This seems to contribute to higher genomic fidelity and prediction of therapeutic responses. Indeed, engrafted PDXs in immunocompromised mice show good correlation with HSNCC aggressiveness and related survival. Moreover, their usefulness concerning the identification of drug targets has been established in several works that use a large collection of PDX models of HNSCC to demonstrate the efficacy of different therapeutic strategies for certain patients [14, 15].

Cell lines derived from PDXs allow the expansion of stable cell lines that can be used as predictive preclinical models for screening assays while reducing variability, animal usage and study costs. Low-passage cultures of these cells maintain the biological properties of the original tumour. In a recent study by Khalil et al., an agonist of the nuclear receptor NR2F1 was reported to arrest cell growth in an HNSCC PDX line [16], and was suggested as a promising pharmacological alternative.

Added to the typical limitations of PDX models, PDX-derived 2D cultures should not be used after more than 10 passages to avoid extensive genetic and epigenetic differences [15].

### In vitro 3D models

Drug screening and molecular biology assays on monolayer cell cultures are still popular methods in cancer research. However, 3D cell culture strategies reflect tumour tissue architecture to a better extent. They include spheroids, which are cultured free-floating aggregates derived from cancer cells; organoids, which are miniature versions of organs derived from stem cells, and microfluidic systems.

Spheroids are formed by spontaneous aggregation of different cells that can have different sources, giving rise to multicellular tumour spheroids (MCTS) or tumour-derived spheroids (TDS). MCTS only contain tumour cells, are clonal and grow easily into large cultures, but they do not resemble the histology of the original tumour. In contrast, TDS are not only enriched with tumour cells but also with stem cancer cells, providing a heterogeneous tumour environment [17]. Spheroids may be obtained using different protocols: some using scaffolds or microbeads to induce cell aggregation, mostly used in regenerative medicine, and some that do not need a scaffold. They may use suspension cultures, ultra-low-attachment plates, hanging drop and microtechnology platforms, and are far more economic and simpler than other types of 3D models [18, 19].

In a recent study, Kochanek et al. used HNSCC MCTS from five human HNSCC cell lines to evaluate the effects of 19 anticancer agents. Among them, five chemotherapy drugs have been approved for head and neck cancer: methotrexate, 5-FU, bleomycin, cisplatin and docetaxel; also, the EGFR tyrosine kinase inhibitors gefitinib and erlotinib were used as surrogates for cetuximab, or dactolisib, a dual PI3K and mTOR inhibitor. To assess the performance advantage of MCTS versus 2D cultures, they tested the set of 19 drugs with established 2D monolayer growth inhibition assays using the same HNSCC cell lines. They found that MCTS cultures had slower cell proliferation and growth rates, like solid tumour growth in vivo. Also, MCTS cultures showed higher resistance and lower drug penetration and distribution in most of the tested drugs increasing the resistance of some of them. Using this system, MCTS drug responses could be stratified into high, intermediate, and low impact tiers with a cumulative multiparameter drug impact score, maximising the usefulness of MCTS tumour cultures to establish models of resistance found in HNSCC patients [20].

The main advantages achieved through organoid technology are the preservation of the in vivo 3D architecture and the proliferation of heterogeneous cell types of the tumour microenvironment. Therefore, they are postulated as novel in vitro models for drug testing, and monitoring the response to therapy. They were first developed by Köpf-Maier and can be formed by pluripotent embryonic or induced stem cells (PSC) or by adult stem cells (ASC) [21]. Embryonic or induced stem cells differentiate in several cell subsets to generate the appropriate distribution of mesenchymal, epithelial and endothelial cells which assure the essential tissue-specific biological processes are working in the model. However, the generation process of these organoids is complex, typically slow, and requires the addition of several differentiation factors. In the case of ASC organoids, adult stem cells from tissue compartments with regenerative capacity are used instead. They are simpler and more homogenous for long-term expansion. In both cases, single-cell suspensions usually require large droplets of 3D Matrigel as support [17].

Sawant et al. generated a panel of 31 HNSCC organoids that evidenced that tongue tumorigenesis can be developed and reproduced in vitro. They identified cancer-associated fibroblasts as a key population for mimicking HNSCC pathogenesis and for the regulation of the epithelial thickness, cell proliferation, differentiation and maintenance of junctions in in vitro grown tissues [22].

In particular, HNSCC organoids from oral mucosal or malignant tongue tissue reproduce genetically, histologically and functionally the disease [22, 23].

Despite different establishment success rates among 3D models, there is a consensus of their promising potential for in vitro drug testing. Indeed, several studies demonstrated that, in oncological drug testing, spheroid models derived from human tumours outperform the in vitro gold standard [24].

The advances in microfluidics technologies have allowed to take advantage of their potential in several fields. Particularly, in cancer and immunotherapy research, it has offered the possibility of controlling the culture environment in small compartments, which represents a promising improvement for the study of the mechanisms involved in the interaction between tumour and immune systems [25]. We find an example of this approach applied to HNSCC research in Hattersley et al, work, where they demonstrate the preservation of the original tissue architecture inside their microfluidic device [26]. However, this system has disadvantages, as it is quite expensive, technically challenging and more time-consuming, compared with other in vitro systems [27].

Even though these 3D models have improved in vivo resemblance, they are conceptually limited to the process inherent to the tumour microenvironment, therefore they cannot mimic the complete physiological regulation that also influences pathogenesis and drug efficiency, particularly problematic for new generation drugs involving remote cells, as it is the case of the immunological system in immunotherapy. These in vitro model limitations emphasise the importance of the development of specific in vivo models for the study of HNSCC.

## HNSCC in vivo models

Although in vitro models have demonstrated to be very useful in HNSCC preclinical research, in vivo animal models are essential to fully understand the mechanisms and molecular events happening during HNSCC initiation and progression in their specific environment. To this purpose, different approaches have been developed, including carcinogen-induced HNSCC models, transgenic animals and transplantable xenograft models.

The range of animal model species that have been the subject of study for preclinical models of HNC go from domestic animals with spontaneous cancers, because of their similarities with human HNSCC [28], to models based on carcinogenesis induced in the cheek pouch of hamsters, which have been historical models for chemoprevention studies [29], although they include species more recently found useful for this purpose, like zebrafish.

Although spontaneous lesions found in domestic dogs and cats have provided interesting information about drug pharmacokinetics and diagnostics in HNSCC [30, 31], and may show advantages because the tumorigenesis is similar to the human one, these models' result less convenient because of the rare availability of species-specific reagents, and the still questionable extrapolation to human disease and drug metabolism mechanisms [32, 33].

There are numerous studies of carcinogenesis [34] and cancer chemoprevention [35] that use hamsters as a model. Lesions are induced by directly applying carcinogens to the mucosal cheek pouch, and the development of the disease has been found to be similar to the human counterpart. However, the main disadvantage is precisely the use of an anatomical structure that does not exist in humans, and that could be immunocompromised, so not all immune interactions are considered. In addition, this model fails to generate metastatic lesions [29, 36].

Zebrafish larvae have also been used as a model for cancer due to the conveniently reduced time and costs needed to develop a high-scale experiment [37]. Indeed, their small size and their prolific and short life cycle allow to complete experiments in a short time. The larvae of these fish are translucent, which is very convenient for in vivo imaging; they are also able to absorb small compounds. With these characteristics, they have proven particularly useful for drug discovery [38] and the study of the mechanisms of cell proliferation, angiogenesis and metastasis [39, 40].

More recently, zebrafish models have been introduced to cancer studies as xenograft models, generated by microinjection of patient-derived cell suspensions (100–500 cells per embryo are normally enough [41]) into the embryonic perivitelline space. Particularly in oral cancer, Wen et al. unveiled the role of MMP-9 in OSCC cell invasion and metastasis, proposing its potential as therapeutic target [42], Nicoli et al. developed a PCR-based method for drug screening that is comparable to imaging regarding performance (and more convenient) in zebrafish larvae models [43]. Mohapatra et al. demonstrated the therapeutic effects of the genetic inhibitor (CMTM6KD) and the pharmacological inhibitor (CX-5461) in chemoresistant OSCC [44]. Finally, Hujanen et al. used a zebrafish model to reproduce the development of tumour-related neoangiogenesis in HNSCC [45]. However, further studies are needed to warrant an adequate extrapolation of the characterised processes in zebrafish to the human system.

Since mice are the most suitable choice compared to other animal models given their standardised, controlled, and extended use in research, they will be the focus of this section.

### PDX animal models

This animal model, firstly described in 1969 [46], is generated by implanting a xenograft from a tumour cell line or a patient tumour biopsy, normally subcutaneously, in a recipient mouse [47].

The engraftment success rate may vary depending on the tumour histology, collection methods or choice of mouse strain. The development of immune-deficient mouse strains to be used as PDXs recipients has facilitated the success of these implants. The NOD/SCID/IL-2Rγ^−/−^ (NSG) mouse, which lacks mature T and B cells, is the most commonly used immunodeficient mouse to produce PDXs, as researchers have found a high rate of engraftment and tumour growth; also, tumour regression is reduced when using this strain [15].

In a study by Yen et al, several PDX models of oral squamous cell carcinoma (OSCC) were established and pair-characterised with their primary tumours by WES and RNA-seq, showing that PDX strains are able to maintain most of the genetic mutations from the primary tumour. This study, which is however very recent and has yet to report the validation cohort, has also offered a gene expression profile of five genes (*MMP1*, *FBLN5*, *COL5A3*, *BGN* and *LOXL1*) that can predict successful xenograft engraftment [48].

The major advantage of PDX models is that the implant preserves the original heterogeneity and molecular identity of the human tumour to a major extent than the in vitro systems. This is very useful for the evaluation of the response to therapies; indeed, they have proved valuable for the clinical drug screening of mucinous salivary adenocarcinoma and other head and neck tumours. Panaccione et al. developed a mucinous adenocarcinoma PDX model which exome sequencing pointed out potential drivers of invasion and metastases, including R213X *TP53* and G13D *KRAS* mutations, both previously described in other cancers [49].

There are a few groups that have tried to generate PDXs from CTCs, with the aim to minimise the use of invasive techniques such as tissue biopsy. However, the rarity and high variability of CTCs according to the primary site and disease stage, makes generating HNSCC PDXs from CTCs still technically challenging [50]. Despite this, the characterisation at single-cell level of lung cancer CTCs indicates their potential use for drug response testing and micrometastasis identification. Primary NSCLC tumour patient-derived xenograft models established by Suvilesh et al. proved to generate in the mouse circulating tumour cells (CTCs) populations that resembled those isolated from the patient. By liquid biopsy molecular profiling, they demonstrated that these cells also represented the primary tumour, both pathologically and phenotypically, associating the epidermal growth factor receptor (EGFR) pathway with aggressive tumour growth [51].

Regarding the general drawbacks of PDXs, it is important to emphasise that their establishment and maintenance are time-consuming, expensive and laborious. In addition, the clones that are selected and grown in the mouse may not fully resemble the behaviour of the original tumour, and in those cases the observations are not easily extrapolated to the human disease. Numerous traditional studies based in murine models allowed the identification of antitumor drugs that were actually triggering massive DNA damage in human bone marrow, and some compounds like brefeldins and minor groove DNA binders, which have an effective antitumor effect in human, instead show no effect in mice. In a similar manner, some drugs may have significant antitumor effects in xenograft models, but they show no benefit when used in human [52, 53].

Another relevant issue is the requirement to use immunodeficient mouse strains in order to ensure implant success, which has obvious limitations in achieving a resemblance with the human pathologic environment since it oversees the important involvement of the immune system in tumorigenesis, tumour progression and therapy response in HNSCC. The generation of humanised mice being incorporated to these studies has led to promising advances in this sense. The relevance of the immune implications in mouse PDX models are amply discussed by Rossa et al. [54]. To generate this model, human hematopoietic stem cells are transplanted into immunodeficient mice in order to restore a “humanised” immune system in the recipient mouse before the PDX transplantation. Recently, a protocol for this method has been published by Fu et al., where patient’s bone marrow cells are used to humanise the mouse recipients avoiding the risk for HLA mismatch. These mice were then successfully transplanted with HLA-A2 + HNSCC tumours, and human T cells were found successfully infiltrated afterwards into an autologous tumour, allowing to study the interaction between immune cells and tumour in a humanised context [55, 56].

Finally, and in line with the scarcity of cell lines derived from HPV + HNSCC tumours, other caveat of this system is the lack of standardised protocols for the engraftment of HPV + tumours, which so far is largely unsuccessful. Few groups have investigated the HPV status in PDXs, such as Kimple et al., who identified 6 HPV + PDXs with p16 staining in 22 HSNCC PDXs [57]. Moreover, EB virus-positive lymphoma contamination results in a lower rate of success [58]. Consequently, most PDXs are sourced from HPV- human oral squamous carcinoma from the tongue, soft palate, or floor of the mouth, and, less frequently, from the oropharynx or hypopharynx [57]. Addressing this limitation should be prioritised given the recent rise of HNSCC cases associated with HPV infection.

At this point, it must be addressed that PDX cells undergo what is called genomic evolution during their derivation and clonal expansion. Genomic evolution is the alteration of the tumour genome over time due to selection or genetic drift, and it has been associated with tumour progression and treatment resistance. Several studies strongly suggest that both pre-existing and de novo mutations occurring during propagation of the PDX, which include copy number variations, confer a high genomic instability to their cells, and this phenomenon could be reflected in phenotypic consequences [59–61]. Therefore, the considerable rates of these alterations caused by the genomic evolution must be considered in order to properly apply these cancer models. In this sense, Li et al. performed a genomic and clinical characterisation of HNSCC tumours from The Cancer Genome Atlas. They established that the co-occurring somatic mutations are significantly less frequent in HNSCC that undergo neutral tumour evolution, and associated this event with a more active immune response, more efficient immunotherapy and improved survival of patients [62].

### Syngeneic models

To generate syngeneic mouse models, tumour tissues or cells from mice are transplanted into another mouse with similar genetic background, allowing to keep the full immune capabilities of the receptor. Therefore, the normal immunologic behaviour against the tumour can be reproduced, and the observed mechanisms resemble the human setting to a higher extent. This model is more appropriate for the study of those processes requiring the interaction between the tumour and the host environment, not only immune responses, but also stromal signalling, angiogenesis and metastasis.

Various studies have generated syngeneic models by orthotopic transplantation in the floor of the mouse mouth [63], and several works have shown how the generated lesions offer morphologies compatible with dysplasia, hyperplasia, in situ carcinoma and invasive squamous carcinoma [64, 65]. The destructive nature of the disease is reproduced to such degree that these syngeneic tumour cells are preferably placed subcutaneously in the flank of mice. The model that has been most frequently applied consists of the subcutaneous implantation of the SCC VII/SF cell line in the C3H/HeJ mice [36] and it has been used for a broad spectrum of assays, especially for the evaluation of new chemo- and immunotherapies [66]. However, this specific system offers a poor ability to replicate human disease.

Fu et al. developed a HNSCC tumorigenicity‑enhanced cell line (JC1‑2) from carcinogen-induced lesions. JC1‑2 cells were able to trigger the growth of syngeneic ‘inflamed tumours’ in immunocompetent C57BL/6 mice, in contrast to previous cell lines that could only generate tumours in nude mice. In addition, this model demonstrated microsatellite stability and responsive immune mechanisms, proving its usefulness to better understand the immune microenvironment in HNSCC and the potential to validate new immunotherapy targets [67].

Pain is often the first and most severe symptom characterising oral cancers and it can appear in different forms and intensities, especially in cases where perineural invasion or lymph node metastasis occurs. There is evidence of a cross-activation mechanism between the tumour and the peripheral nerves. Because the glial and immune modulation around the tumour microenvironment is a key factor for the pathobiology of pain mechanisms, the immunocompetent syngeneic animal model is particularly useful to follow cancer progression and pain signalling in HNSCC. There is a recent and interesting review on this and other preclinical models of head and neck cancers in pain research by Ye et al. [68].

### Carcinogen-induced models

HNSCC carcinogen models may be the best approach to mimic the human clinical disease, which is normally originated by the long-term exposure to low doses of carcinogens. In chemically induced cancer models, lesions are generated by the application of a potent chemical carcinogen. Exposure to 4-Nitroquinolone oxide (4NQO) is an alternative for tobacco exposure in animal models of human oral squamous carcinogenesis and has been broadly used for investigating the effects of anti-tumour drugs [69]. This carcinogen has a high rate of success in generating multiple neoplastic lesions in mouse and rat that show histological changes and pathological behaviours, also in an immunocompetent environment, that mimic oral cancer development in humans, and the method was standardised by Tang et al. in 2004 [70].

A different model in use is generated through the administration of dimethyl-1,2,benzanthracene (DMBA). It has been described as carcinogenic chemical in hamster and mouse models [71].

The carcinogen-induced model has the advantage of allowing the observation of the tumour generation from its origin with a molecular, histological and immunological behaviour that resembles the human HNSCC clinical features. It is especially useful for the study of carcinogenic mechanisms and the evaluation of the immunotherapeutic value of newly developed therapies in the different stages of the disease.

However, this method requires a long term of study- up to 40 weeks- to fully evaluate the development of the effects, and therefore it is more tedious to reproduce it. To address this limitation, Wang et al. propose an alternative syngeneic mouse model that uses 4NQO-derived cell line-induced tongue tumour xenografts as a potential shortcut. In this model, lymph nodes start to be invaded after only 2 days of implantation, and metastasis is observed after 8 days. Moreover, mouse HNSCC lesions treated with in situ anti-CTLA4 showed similar immune infiltration and response rates than those induced by anti-PD-1 treatment in human HNSCC, so it could be used to identify response biomarkers that are relevant for patients [72].

### Transgenic models

An important number of potential driver genes involved in HNSCC development have been discovered by molecular profiling studies [8]. Thus, genetically engineered transgenic mouse models (GEM) may provide information about the biological effects of specific mutations in a controlled genetic background. GEM models are generated to express oncogenes or tumour suppressor genes in immunocompetent systems, normally through the alteration of its promoter regulation. In this sense, this is a more realistic model for studying the molecular mechanisms of cancer origin, and it is particularly promising in HNSCC research because of their potential to also recapitulate tumour progression. The use of this method to generate models of head and neck tumours is relatively recent [36]; and includes both the generation of endogenous mutants through knockout or knock-in technologies, and conditional GEM models where the mutation can be induced at specific sites and times.

Mutations in *K-ras* have been the most common modifications used to generate HNSCC genetically engineered models through this system. In the *SL-KrasG12D* mouse, *K-rasG12D* is overexpressed in the oral epithelium of mice, regulated through different keratin promoters [73, 74]. However, this single-transgene model developed oral and tongue papillomas in very few cases and only after eight weeks of treatment. In contrast, double-transgene models such as *KrasG12/TP53*^*del/del*^*, KrasG12/KLF4*^*del/del*^ or *KrasG12/Smad4*^*wt/del*^ were more successful in generating tongue carcinomas as early as two weeks after induction with higher prevalence in oral tumour formation [75–77]. However, it must be observed that the frequency of the driver mutations in HNSCC patients is low. *HRAS* and *KRAS* mutations are detected in only 0.2%, and homozygous deletions of *KLF4* and *SMAD4* in 4% of cases. On the other hand, 30–35% of primary HNSCC tumours present *SMAD4* loss of heterozygosity or high intratumoral heterogeneity, constituting a good candidate oncogene for these models. Indeed, *Smad4*^*del/del*^ mice have shown increased genomic instability and decreased expression and function of genes encoding proteins in the Fanconi anaemia/BRCA DNA repair pathway, which has been linked to human HNSCC susceptibility. Using this model, Hernandez et al. spotted a marked heterogeneity in *SMAD4* deletions in HNSCC patients, both inter- and intra-tumour, and demonstrated that the *SMAD4* FISH assay could be used as diagnosis tool to assess chromosomal loss which could be associated with prognosis in these patients [78].

In case of HPV + oral tumours, transgenic animal models can be generated by genetically engineering them to express the HPV oncogenes E6 and E7 [79]. More recently, a more efficient model has been designed to induce spontaneous HPV + tumours using plasmids encoding the HPV oncogenes and a synthetic transposable element together with a transposase that randomly integrates them in the host genome. This model succeeded to obtain the persistent expression of HPV16-related genes and to mimic the stages in cancer progression from initiation to local invasion and metastasis rapidly [80].

A general limitation of the GEM models is that the genomic engineering needs a sophisticated fine-tuning and even with that, given the sporadic nature of these lesions, the mutations generally present low tumour penetrance and tumours are generated with low frequency, so the outcome of the experiments is usually unpredictable. Another drawback of this model type, in the case of endogenous GEM animals, is that all tissues are expected to present the engineered genomic modification. This can interfere with other physiological processes and affect the fidelity of the model for the tumour disease. Finally, several bioethical considerations regarding the genetic modification of animals must also be considered when selecting this method.

Recently, several studies have used a combination of genetic modifications and chemical carcinogens to develop HNSCC mouse models that are showing high efficiency and faster results. A remarkable 100% efficiency has been seen when applying 4NQO to *XPA*^−/−^; *p53*^+/−^ mice, which develop tumours in 25 weeks, compared with 50 weeks without the carcinogen [81] or to *PIK3CA*-GEMM mice. In this case, PIK3CA overexpression was restricted to the head and neck, and tumours were generated in 100% of mice after 10–12 months [82].

## Concluding remarks

The biological and clinical heterogeneity of the different anatomical locations where HNSCC can appear, has made it difficult to obtain good preclinical models. Approaches using HNSCC cell lines constitute a very affordable method to understand the molecular biology of tumours and to screen novel drugs before their introduction into clinical use. However, these models become inefficient to predict the entire complex behaviour of the tumour in an in vivo system and are not appropriate for customising the treatment to individual cancer patients. The development of technologies using 3D cultures, although currently expensive, could be a more promising way of achieving the complexity that better resembles the in vivo situation, while remaining under more controlled conditions and allowing high-throughput drug screening. However, these models will never be a faithful reproduction of an in vivo environment.

Traditionally, the development and evaluation of novel therapies have relied on orthotopic mouse models, which show higher similarity to human HNSCC, in particular at the disease initiation stage. However, not a single murine model meets the ideal features for both the study of the HNSCC pathogenesis and the prediction of the response to therapy. On the one hand, carcinogenesis models have yielded useful data for the study of chemoprevention but have limitations in case of other purposes. On the other hand, genetically modified mice are useful for studies focused on genetic mechanisms but also have several limitations regarding low tumour penetrance and outcome uncertainty. Recently, there has been a rise in studies that combine the use of carcinogens on transgenic models, which accelerates the growth of tumours, with promising advances. However, there are further technical challenges that remain to be addressed, such the modelling of metastases. In fact, these models are very scarce in the field of HNSCC. There are PDX models that develop metastasis and local bone invasion, but these cannot be set in immunocompetent recipients. The future perspectives are set then in the establishment of new models able to achieve metastasis in genetically modified mice.

In silico approaches, currently in expansion, are promising technologies to study biological systems. Mathematical modelling is an inexpensive and useful tool, as it allows to virtually and preclinically evaluate outcomes in cancer progression and therapy efficacy through the consideration of multiple parameters that could not be easily integrated through conventional wet lab techniques [83]. As a drawback, the risk of overseeing determining factors is high, considering the very complex network of parameters that must be integrated to recreate the system.

In conclusion, despite the ample battery of in vitro and in vivo models of HNSCC that are already available, the particular heterogeneity of this type of tumour and the general limitations of the current models, make it still necessary to refine the current methods. Further efforts are required to generate and optimise reliable preclinical models to achieve a better understanding of the molecular, cellular, and immunological mechanisms involved in the initiation, treatment resistance and progression of the disease. The availability of models that most efficiently predict the clinical outcomes of disease and treatment would also facilitate the discovery of biomarkers of disease and response and in turn, improve the lives of the patients.

## Data Availability

Not applicable.
